# Editorial: Neuroplasticity and imaging methods in rehabilitation

**DOI:** 10.3389/fnhum.2024.1540391

**Published:** 2024-12-20

**Authors:** Ilan Laufer, Alark Joshi

**Affiliations:** ^1^Department of Industrial Engineering and Management, Ariel University, Ariel, Israel; ^2^Department of Computer Science, University of San Francisco, San Francisco, CA, United States

**Keywords:** neuroplasticity, cognitive rehabilitation, imaging technique, telerehabilitation, white matter (WM), brain connectivity, personalized therapy

Neuroplasticity, the ability of the brain to adapt and reorganize, is a foundation for recovery and rehabilitation. Advances in imaging techniques have revolutionized our understanding of these processes, providing tools to explore the underlying mechanisms and inform practical strategies for improving patient outcomes. This Research Topic highlights key investigations into how imaging methods can advance the science and practice of rehabilitation.

## Exploring neuroplasticity with imaging

The contributions in this collection reflect diverse approaches to leveraging imaging techniques in rehabilitation research. Caminiti et al. design a protocol to assess the effectiveness of cognitive telerehabilitation methods for mild cognitive impairment, with a focus on evaluating changes in brain connectivity and cognitive performance over multiple follow-up intervals. Kirby et al. explore how sex differences shape white matter neuroplasticity during motor learning. The study shows that females exhibit more pronounced structural adaptations in white matter compared to males, despite similar improvements in motor performance. These findings highlight the importance of considering biological differences when developing personalized rehabilitation strategies. Lotze highlights the importance of performance monitoring in longitudinal fMRI studies to ensure reliable and interpretable results. By addressing variables such as task precision, timing, and physiological responses, the study emphasizes strategies for minimizing confounding factors. These approaches improve the accuracy of imaging data and support the development of more robust neurorehabilitation interventions. Silva et al. investigate the use of Galvanic Vestibular Stimulation (GVS) to improve balance in patients with HTLV-1-associated myelopathy (HAM). The study reports significant short-term improvements in mobility and balance, with patients showing faster mobility (TUG test) and better balance (Berg Balance Scale scores) following the 12-week intervention. However, these gains diminished over time without continued stimulation, suggesting that ongoing use of GVS or home-based devices may be necessary to maintain long-term benefits.

## Broad themes and insights

These studies highlight the complexity of neuroplasticity research and its practical implications for rehabilitation. One of the main takeaways is the importance of personalizing interventions, as demonstrated by Kirby et al. and Caminiti et al.. They show how interventions can be tailored to meet individual needs, whether by considering biological differences or using targeted cognitive telerehabilitation methods. Another critical aspect is the methodological advancements, with Lotze's work on refining performance monitoring in fMRI studies to ensure more reliable and accurate results. Silva et al. also emphasize the importance of long-term strategies for maintaining therapeutic benefits, particularly in chronic conditions like HTLV-1-associated myelopathy, underlining the need for continuous stimulation to sustain progress.

## Future directions in rehabilitation science

The studies in this collection point to promising avenues for advancing rehabilitation science. Caminiti et al. focus on evaluating cognitive telerehabilitation methods for MCI, aiming to understand their effectiveness in improving brain connectivity and cognitive performance. The importance of biological differences, as highlighted by Kirby et al., suggests that incorporating such factors can lead to more effective, personalized therapies. Lotze's work shows how refining imaging methodologies improves the precision of neuroplasticity studies, further strengthening the foundation of rehabilitation research. Finally, Silva et al. offer insights into the need for continued stimulation to maintain therapeutic gains, pointing to the potential benefits of home-based devices or sustained interventions.

